# Unassigning bacterial species for microbiome studies

**DOI:** 10.1128/msystems.00515-24

**Published:** 2024-06-24

**Authors:** Ceylan Tanes, Vincent Tu, Scott Daniel, Kyle Bittinger

**Affiliations:** 1 Division of Gastroenterology, Hepatology, and Nutrition, Children’s Hospital of Philadelphia, Philadelphia, Pennsylvania, USA; University of North Carolina at Charlotte, Charlotte, North Carolina, USA

**Keywords:** microbiome, 16S rRNA marker gene, taxonomic assignment, species-level

## Abstract

The method of 16S rRNA marker gene sequencing has fueled microbiome research and continues to be relevant. A perceived weakness of the method is that taxonomic assignments are not possible to make at the rank of species. We show that by working to *rule out* bacterial or archaeal species membership, we can provide an answer that is more accurate and useful. The Unassigner software operates on 16S rRNA marker gene data and computes a rule-out probability for species membership using a beta-binomial distribution. We demonstrate that our approach is accurate based on full-genome comparisons. Our method is consistent with existing approaches and dramatically improves on them based on the percentage of reads it can associate with a species in a sample. The software is available at https://github.com/PennChopMicrobiomeProgram/unassigner.

**IMPORTANCE:**

While existing methods do not provide reliable species-level assignments for 16S rRNA marker gene data, the Unassigner software solves this problem by ruling out species membership, allowing researchers to reason at the species level.

## INTRODUCTION

The method of 16S rRNA marker gene sequencing is well established as an approach to profile bacterial communities in microbiome studies. Over 55,000 publications mention 16S gene sequencing in the current era of microbiome research, 2010–2022. Although shotgun metagenomic sequencing has gained in popularity as a competing approach to characterize bacterial communities, marker gene sequencing continues to be the best option for microbial environments with high levels of host DNA (1). A perceived weakness of 16S rRNA marker gene sequencing is the inability to make taxonomic assignments at the rank of species, a limitation not inherent to shotgun metagenomic sequencing, especially in host-associated biomes (2).

16S rRNA gene sequencing is also employed by microbiologists who isolate bacteria and archaea to establish new species. To establish a new species, researchers must demonstrate that a strain is sufficiently different from the type strains of existing bacterial species. Type strains define established species and, thus, inhabit a role of unique importance in bacterial taxonomy. Members of a bacterial species are genomically and phenotypically similar to the type strain; otherwise, they are eligible to be a new species. In 1994, Stackebrandt and Goebel (3) observed that, while 16S gene similarity may be high (>97.5%) between members of different species, 16S gene similarity was seldom low between organisms of the same species. According to the current recommendations for establishing new species, researchers may examine 16S gene similarity to existing type strains and conclude that a strain is not a member of an existing species if the 16S gene similarity is less than 97.5% (4).

Here, we apply the procedure outlined above to 16S rRNA marker gene sequencing data. Although we cannot definitively assign bacterial species based on 16S gene data, we are able to rule out many or all bacterial species as potential assignments with high confidence. Thus, by changing the question slightly, we arrive at results that are nearly as useful as “real” species assignment in 16S marker gene studies. Our software, the Unassigner, provides a rule-out probability for bacterial species and eliminates all but a small number of compatible species for each read in 16S marker gene sequencing data.

## RESULTS

### Description of the method

The underlying philosophy behind our approach is to follow the process used by microbiologists to define new bacterial species in the literature. Each bacterial species is represented by a type strain, which represents the species. When establishing a new bacterial species, microbiologists compare new isolates to the type strains of existing species. Microbiologists cannot definitively assign a strain to an existing species using only the 16S rRNA gene sequence, but they may use a 97.5% gene similarity threshold to rule out the compatibility of a strain with the type strains of existing species.

Our software automates this process for high-throughput marker gene sequencing data, calculating the probability that existing bacterial species can be ruled out as the assignment for each query sequence. Often, all known species are ruled out. However, when the sequence is consistent with a named species, our software outputs the species name and the rule-out probability. When more than one species is consistent with the sequence, our software outputs all the species that cannot be ruled out. Hence, the software is built to *unassign*, rather than assign, bacterial species to 16S rRNA marker gene sequences.

**Fig 1:**
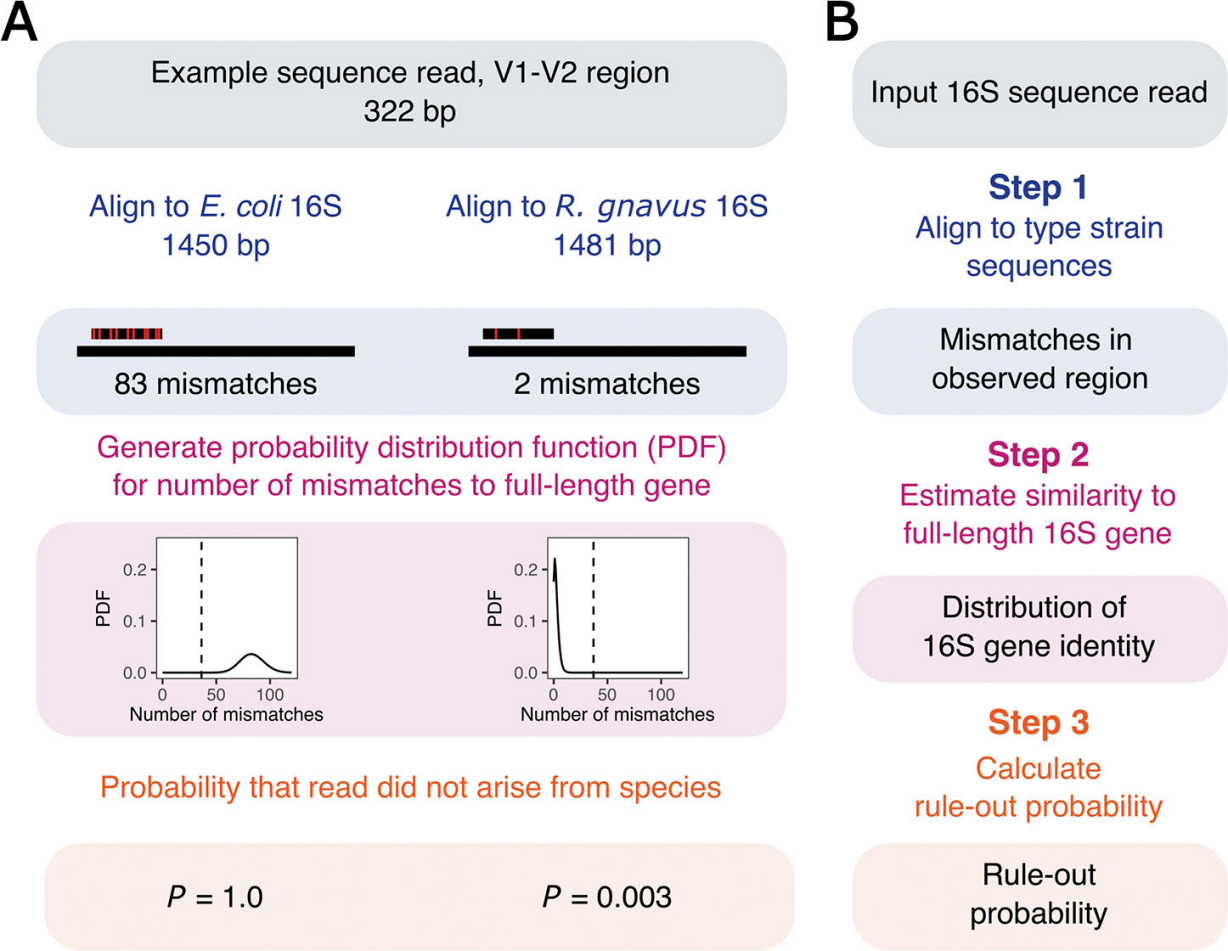
Overview of the method. (**A**) Example of rule-out probability calculation for one sequence read of 322 bp and two potential species exclusions. For *E. coli*, the example read has 82 mismatches in the aligned region. The software then computes a probability distribution function (PDF) for the total number of mismatches across the entire gene, determines that no possible outcome is consistent with the read arising from *E. coli*, and returns a rule-out probability of 1.0. For *R. gnavus*, the sequence read has two mismatches in the aligned region. The PDF for the total number of mismatches is mostly consistent with the read arising from *R. gnavus*, and thus, the rule-out probability is low. The vertical dotted line represents the number of total mismatches corresponding to 97.5% percent identity. The *x* axis represents the number of mismatches in the unobserved region of the 16S rRNA gene. The beta binomial distribution on the left is generated with the priors alpha of 83.5 and beta of 239.5. The distribution on the right is generated with the priors alpha of 2.5 and beta of 320.5. (**B**) Diagram of the steps taken by the unassigner software to calculate the rule-out probability.

As an example, consider a single sequence read from a microbiome study in which the V1-V2 region of the 16S rRNA gene was amplified and sequenced (Fig. 1A). When the read is aligned to the 16S gene sequence of *Escherichia coli*, we find that the identity is 70%, which is low enough to definitively rule out *E. coli* as the species from which the read derived. When the read is aligned to the 16S gene sequence of *Ruminococcus gnavus*, we find only two mismatches in 322 base pairs, or 99.4% identity. Next, we generate a probability distribution for the number of mismatches across the entire 16S gene, using the information from the short region that was sequenced. Finally, we use the number of mismatches over the entire gene to calculate the probability that *R. gnavus* might be ruled out as the species from which the read originated. In our example, the rule-out probability is low, approximately 0.003. While we have not definitively assigned the read to *R. gnavus*, we have ruled out all other established species in this hypothetical database of two sequences. Either the read arose from *R. gnavus*, or it arose from a yet-undiscovered, yet-unnamed bacterial species with a 16S gene sequence that is very similar to *R. gnavus*.

The software performs its work in three steps (Fig. 1B). In step 1, we align input sequence reads to a database of full-length 16S sequences from bacterial type strains. The database is derived from the Living Tree Project (5, 6) and includes type strain sequences for 19,135 named bacterial species and 656 archaeal species. Database search and semi-global alignment is performed by vsearch (7). Species candidates with >90% sequence similarity are evaluated in steps 2 and 3.

In step 2, we estimate the similarity for the full-length 16S gene. Because marker gene sequence reads do not cover the full 16S gene, we employ a probability model to derive a distribution for the total number of mismatches over the full-length gene, given the number of mismatches in the observed region. Our software implements two algorithms for calculating the number of mismatches in the full-length 16S gene. For the simpler algorithm, we assume that the underlying rate of mismatches is constant across the entire gene (constant-mismatch-rate algorithm). This assumption is not generally true; for example, the V1-V3 region is more variable than the V4 region of the gene (8). Regardless, the number of mismatches in the unobserved segments of the gene is straightforward to calculate using the beta-binomial distribution (Supplemental Methods). In the second algorithm, we allow for differences in variability between the observed region of the gene and the unobserved segments (variable-mismatch-rate algorithm). Here, we use Greengenes database (9) to estimate the number of mismatches at each position for each type strain. These are then used to calculate the difference in rates inside and outside the observed region by taking the log ratio of expected mismatched rate between the aligned and unaligned regions (Supplemental Methods).

In step 3, we calculate the rule-out probability using the full-length 16S gene similarity. We have also implemented two algorithms for evaluating the species rule-out probability given the similarity for the full-length 16S gene. In the simpler algorithm, we set a hard threshold to rule out compatibility with an existing species type strain, 97.5% gene similarity by default (hard-threshold algorithm). For the second algorithm, we use the results of a comparison between 16S gene similarity and average nucleotide identity between genomes in RefSeq to evaluate the probability that the full-genome identity is less than species-level, given the 16S information (soft-threshold algorithm, Supplemental Methods).

In the sections that follow, we validate our approach by demonstrating that 16S gene similarity is an accurate predictor for the rule-out probability of assignment to a bacterial species. We next compare our software to an existing approach implemented in DADA2 (10). Finally, we compare the performance of bacterial species unassignment in three different 16S gene regions, evaluating the accuracy and deriving optimal parameters for the various algorithms implemented in our software.

### Validation of the method

For our approach to be viable, we must show that full-length 16S gene similarity can be used to accurately predict the rule-out probability bacterial species membership. The leading sequence-based approach to determining the species of a bacterial genome is average nucleotide identity (ANI), where ANI values above 95% indicate species-level similarity between genomes (11). Here, we use a collection of genomes from NCBI RefSeq to compare predictions from 16S gene similarity to ANI values from whole-genome comparisons.

**Fig 2:**
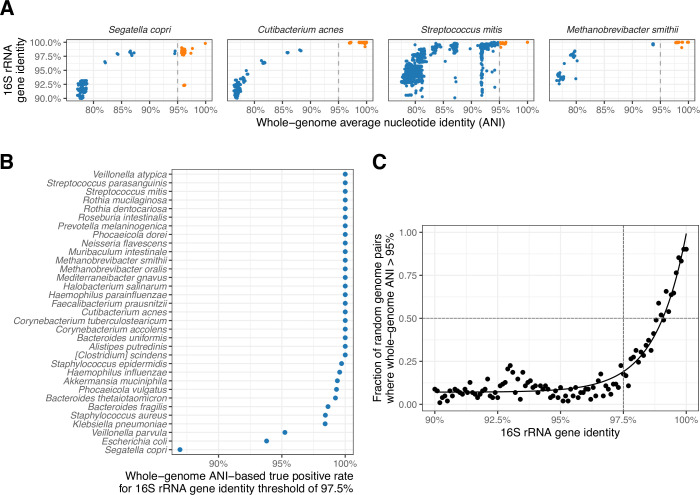
Validation against full-genome comparisons. (**A**) Association between ANI and 16S rRNA gene identity for select species. Orange represents the genomes that would be considered the same species with the ANI threshold of 95%. Blue represents the genomes with greater than 90% 16S rRNA identity but are below the 95% ANI threshold. (**B**) Percentage of genome pairs that are correctly identified by 16S rRNA gene identity, where the correct answer is determined by ANI between the genomes. (**C**) Probability of random genome pairs having species-level ANI as a function of 16S rRNA gene identity.

We selected 30 bacteria and 3 archaea that are commonly found in stool, oral cavity, or skin microbiome and investigated the association between ANI and 16S rRNA alignment percent identity (Fig. 2A; Fig. S1). For each species, 16S similarity and whole-genome ANI values were computed between the type strain and other genomes with >90% 16S gene similarity (Data S1). In an ideal scenario where 16S percent identity is a perfect estimate for ANI, genomes from the indicated species would cluster on the top right corner, with high ANI and high 16S similarity. Conversely, genomes from different species would cluster on the bottom left, with low ANI and low 16S similarity. For species such as *Cutibacterium acnes*, *Roseburia intestinalis*, and *Ruminococcus gnavus*, this pattern held in our analysis. For other species, we recovered the classic pattern observed by Stackebrandt and Goebel (3), where we found genomes with high 16S similarity but low ANI. For species following this pattern, such as *Staphylococcus epidermidis* and *Streptococcus parasanguinus*, genomes compatible with the species by 16S gene identity may or may not be members of the species on the basis of a full-genome comparison. However, genomes with low 16S identity can still be ruled out as species members, which is the quality we rely on to compute the rule-out probability in our software.

We found overall that 16S gene similarity was a reasonable predictor of bacterial species despite some false positive predictions due to high 16S gene identity and low ANI. For the same 33 species, we surveyed the predictive accuracy of species assignment as a function of 16S gene identity. For seven of the species evaluated, the true positive and true negative rates were high for a wide range of 16S gene identity thresholds, between 96% and 99% (Fig. S2A). The accuracy, evaluated by a receiver operating characteristic curve, exceeded areas under the curve (AUC) = 0.9 for all but *Veilonella parvula* among the selected species (Fig. S2B). Furthermore, when we evaluated all the type strains available in RefSeq database, 98% of bacteria and 94% of archaea had AUC greater than 0.9 (Fig. S2C). Because we feel that it may be useful for researchers to investigate the predictive accuracy of 16S gene similarity for their own species of interest, we have made the software for this comparison available in addition to the *unassigner* tool.

We next wished to evaluate the optimal threshold at which 16S gene similarity could be used to predict rule-out probability for bacterial species assignment. To set a hard threshold, we found the value of 16S gene identity rate that captured 95% of the true positives for each species (Fig. 2B). Aside from *Segatella copri* and *E. coli*, none of the selected species required a threshold for 16S gene identity below the standard value of 97.5%. Among all the type strains available in RefSeq, 97% of bacteria and 95% of archaea did not require the standard cutoff to be changed (Fig. S3), making this a safe, if not conservative, threshold for ruling out species membership. Thus, we adopted the value as a default in our software for the hard-threshold algorithm.

For our soft-threshold algorithm, we aggregated our data across all species and estimated the probability of species-level ANI as a function of 16S gene identity (Fig. 2C). The result fits well to an exponential curve, with a half-maximum at 99.1% 16S gene similarity. Our software uses this value as a default when the soft-threshold algorithm is selected.

### Comparison to existing approaches

We evaluated the performance of our software against existing alternatives in the context of previously published 16S rRNA marker gene data sets. To our knowledge, the only other software implementing a method for species-level assignment for 16S marker gene studies is DADA2 (10). In this existing approach, reads are assigned to a species if they are an exact match to sequences in the database with a species annotation. While our approach relies on a relatively small database of type strain sequences, the DADA2 approach uses a larger database and depends critically on non-type strain sequences from a species to be well-represented and correctly annotated. Our approach emphasizes comparison to type strains, in recognition of their central importance to defining bacterial species. In contrast, the DADA2 approach follows the basic strategy used for taxonomic assignment at higher ranks, with tighter thresholds at the species level.

We downloaded 16S rRNA marker gene studies from three human body sites: feces (12), nasal swabs (13), and oropharyngeal swabs (14) and four environmental sites: freshwater sediment, marine, marine sediment, and soil (15–17). After quality control and denoising, we obtained 11,059, 7,380, and 1,504 unique Amplicon Sequence Variants (ASVs) from gut, nasal, and oropharyngeal bacterial communities, respectively. From the environmental samples, we obtained 35,034, 5,279, 8,361, and 7,012 unique ASVs from freshwater sediment, marine, marine sediment, and soil samples, respectively. We then used the Unassigner and DADA2 to classify the representative sequences of the ASVs. In the Unassigner, an ASV was marked as compatible with a named species if the rule-out probability was less than 0.5. This threshold was picked due to the bimodal distribution of the probabilities with peaks around 0 and 1 (Fig. S4). In DADA2, the assignments are done based on 100% similarity.

**Fig 3:**
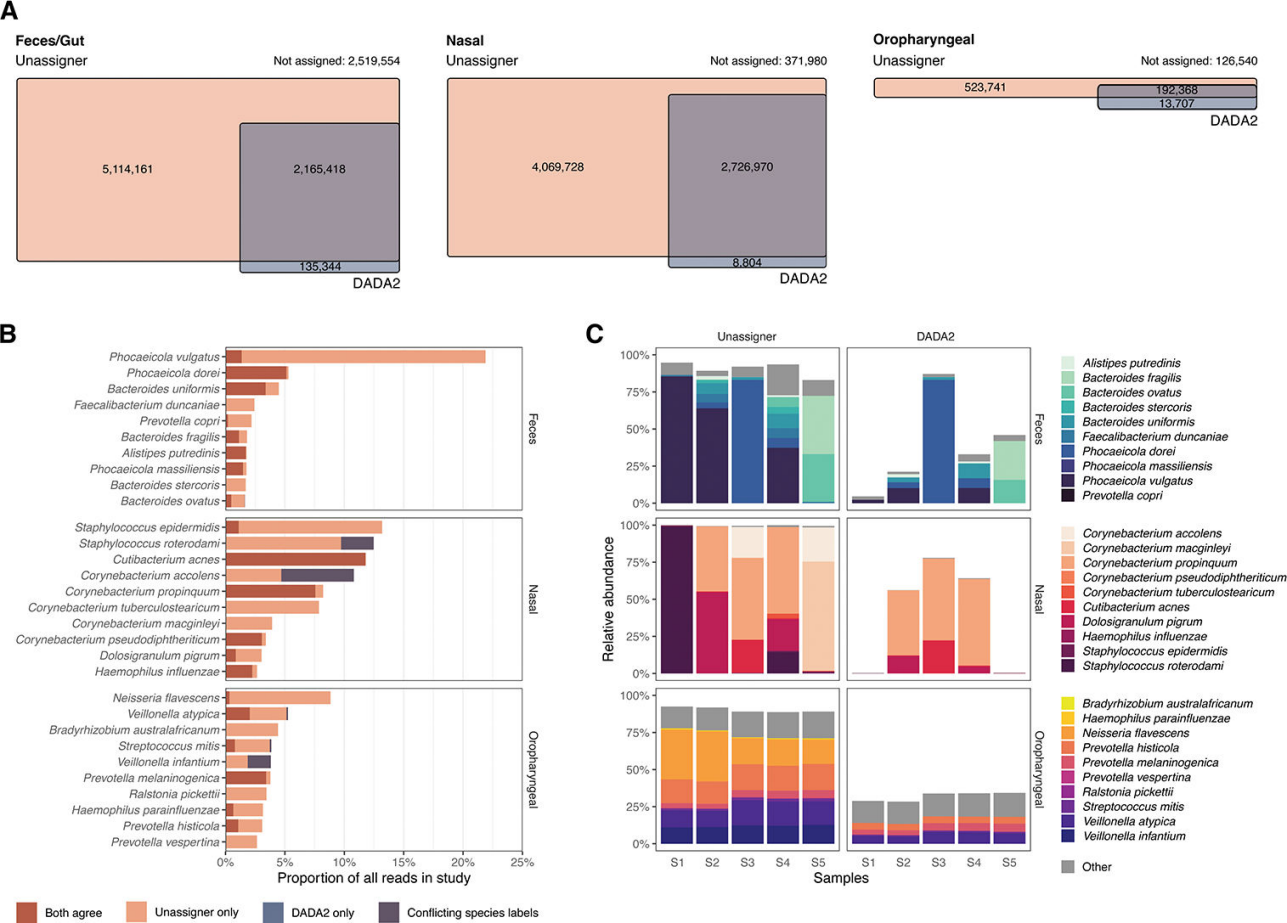
Comparison to existing methods. Results are shown for three example studies of human feces, nasal swabs, and oropharyngeal swabs. (**A**) Venn diagrams of the number of reads assigned to a species with DADA2 or found to be compatible with a species using the unassigner software. The number of reads that could not be attributed to a species by either method is noted above each diagram. (**B**) Species that account for the greatest number of reads in each study. The colors represent the reads that were annotated exclusively by one method, consistently by both methods and the number that had conflicting assignments between methods. (**C**) Species composition of select samples. Uncolored regions represent reads without species inference. From each data set, the five samples with the highest annotation coverage were selected for display.

In all three host-associated data sets, our software identified more species-level annotations than DADA2 (Fig. 3A). Out of all the reads in fecal, nasal, and oropharyngeal samples, 73%, 95%, and 85% of the total reads were compatible with a species using the Unassigner, whereas only 24%, 45%, and 29% of the total reads were assigned with DADA2. Where both methods provided species-level annotations, they generally agreed: only 0.8%, 11%, and 6% of annotations were discordant between the two methods. Only a small fraction of reads were annotated by DADA2 but not by the Unassigner: 1.4%, 0.1%, and 1.5% for the fecal, nasal, and oropharyngeal data sets, respectively. By manual inspection, we observed that in this circumstance, the reads aligned to full-length 16S sequences in the DADA2 reference database that were annotated at the species level but had <97.5% identity to the type strain sequence for the species.

Similar to host-associated sample types, our software identified more species-level annotations in the environmental samples compared to DADA2 software (Fig. S5). Out of all the reads in freshwater sediment, marine, marine sediment, and soil samples, 3%, 7%, 6%, and 31% of the total reads were compatible with a species using Unassigner, whereas only 0.4%, 1.7%, 0.4%, and 2.8% were assigned with DADA2. However, the environmental samples in general had lower compatibility than the samples collected from human body sites. Only 2.9%, 7.7%, 6.2%, and 30.6% of the reads we assigned to a species with either Unassigner or DADA2 software. This is most likely due to the higher diversity of environmental samples and less representation of type strains in the current databases.

For each sample type, we selected 10 species that accounted for the greatest proportion of reads (Fig. 3B). The Unassigner software annotated more 16S reads to prominent species in each body site: *Phocaeicola vulgatus*, *Staphylococcus epidermidis*, and *Neisseria flavecens* for fecal, nasal, and oral samples, respectively. The two methods produced near-identical results for a handful of species, namely *Phocaeicola dorei*, *Cutibacterium acnes*, and *Prevotella melaninogenica*. The most prominent species identified in marine, marine sediment, and soil samples by the Unassigner software were from the Helicobacter genus (Fig. S5b).

Considering the annotations on a sample-by-sample basis, we observed that the Unassigner annotated on average 73%, 94%, and 84% of reads at the species level in fecal, nasal, and oropharyngeal data sets (Fig. 3C). For the environmental samples, the percentages were 5%, 9%, 9%, and 37% for the freshwater sediment, marine, marine sediment, and soil samples, respectively. The overall fraction of reads annotated at the species level varied more from sample to sample using DADA2, relative to our software. The difference in species-level annotations was dramatic for some samples, especially for those with high relative abundance of *P. vulgatus*, *S. epidermidis*, and *Corynebacterium macginleyi*. By manual inspection, we determined that although ASVs for these species aligned to the species type strain sequence with no mismatches, the type strain 16S sequence was not annotated to the species level in the database used with DADA2. Thus, we surmise that the performance of species annotations with DADA2 might be dramatically improved with some revisions to taxonomic annotations in the database. However, our software provides a rule-out probability based on sequence comparison to the species type strain, a relevant quantity that is not estimated by the existing approach.

### Impact of 16S gene region on species inference

For researchers planning 16S rRNA marker gene sequencing experiments, the choice of 16S gene region presents a critical decision. Different regions of the 16S gene have different lengths and variability, qualities which affect the quality of taxonomic assignment downstream. Consequently, we wished to examine the impact of 16S gene region on species-level annotations generated by our software.

**Fig 4:**
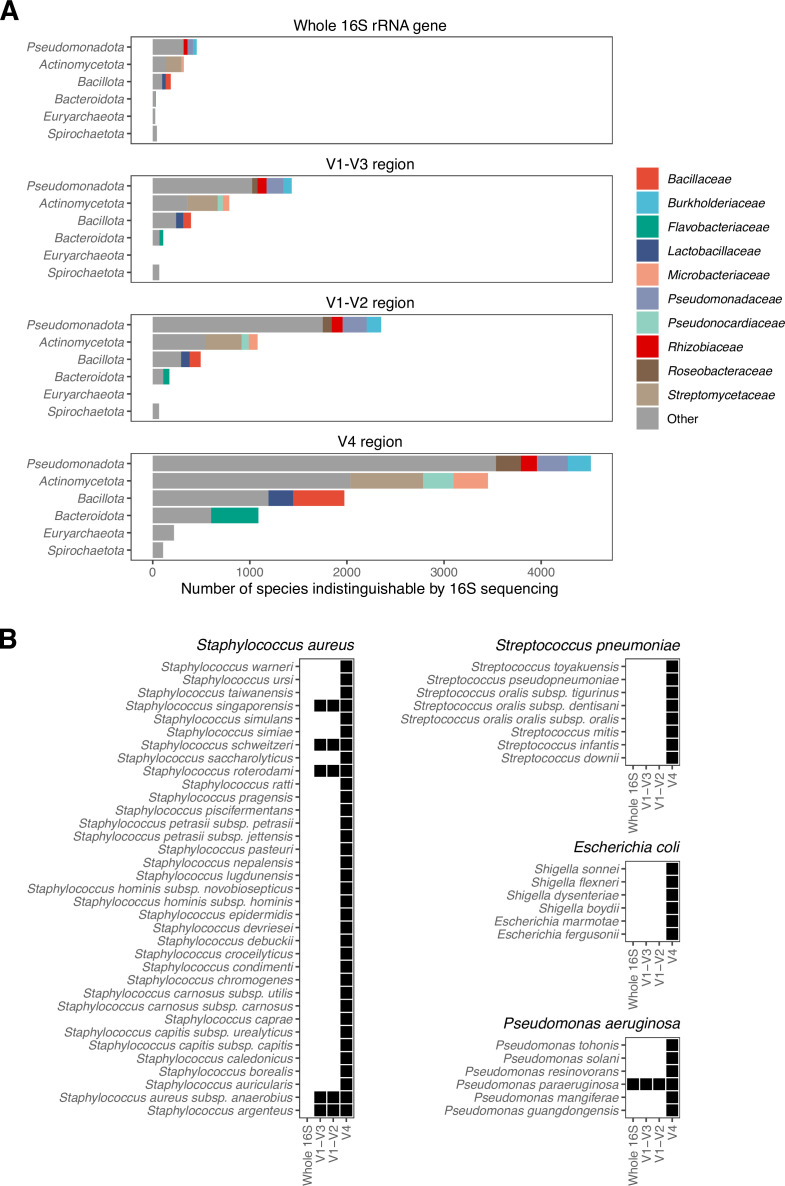
Impact of 16S gene region. (**A**) The number of species with 0 or 1 mismatches to at least one other species in the Living Tree Project (LTP) database for each 16S rRNA gene region. Only the phyla with at least 100 such species in at least one region are shown. (**B**) The list of species with near identical 16S rRNA gene sequences with the select four example species. The four commonly studied species are selected based on the frequency of genomes deposited on NCBI. The black squares represent species with 0 or 1 mutations in the corresponding 16S rRNA gene region relative to the select strain. This would make those species indistinguishable from the select strain.

It is well known that several bacteria species have identical or nearly identical 16S gene sequences. For others, the gene sequence may be identical in one variable region but different over the full-length gene. This phenomenon represents a fundamental limit in the ability of our software, or existing alternatives, to distinguish between closely related species. To identify such inherent limitations of the full-length 16S rRNA gene and compare the ability of variable regions to distinguish closely related species, we aligned type strain sequences and identified species groups with 0 or 1 mismatches (Fig. 4A). Using the full-length gene, we identified 1,112 species that were indistinguishable from at least 1 other species. The greatest number of such species were members of the *Actinomycetota* phylum, in particular, the *Streptomycetaceae*. For the *Bacteroidota* and *Bacillota* phyla, which comprise the majority of the human gut microbiome, only 1% and 5% were indistinguishable using the full-length 16S gene sequence.

We investigated three commonly used variable regions: V1-V2 (18), V1-V3 (19), and V4 (20). The V4 region is used by the Earth Microbiome Project (21) and has seen considerable adoption among marker gene studies in recent years. As the shortest and least variable region of the three, the V4 region had the highest number of species that were indistinguishable from at least one neighbor: 11,917 of 19,791 total named species. The longer and highly variable V1-V2 region had fewer indistinguishable species, 4,395 in all. Sequencing the V1-V3 region requires special 300 bp chemistry on Illumina sequencers, but the additional gene length reduced the number of indistinguishable species to 2,953.

We examined the indistinguishable groups more closely for several species that are especially relevant to the human microbiome (Fig. 4B). *Staphylococcus aureus*, for example, has no indistinguishable species neighbor based on the full-length 16S gene sequence. In the V1-V2 or V1-V3 regions, four species are indistinguishable, whereas 30 additional species are indistinguishable based on the V4 region. Regardless, even a list of 34 species is better than assignment at the genus level—other *Staphylococcus* species important to the human microbiome, such as *S. epidermidis*, cannot be conclusively distinguished from *S. aureus* based on the V4 region but can be using V1-V2 or V1-V3 regions.

We evaluated the performance of the algorithms implemented in the unassigner software by comparing the results from the 16S rRNA regions against the results from the whole 16S rRNA gene (Fig. S6). The receiver operating curves (ROC) show that the AUC for all selected regions are above 0.8 for the default and variable mismatch algorithms. The AUC is greater than 0.9 for all implemented algorithms for the longer V1-V2 and V1-V3 regions.

## DISCUSSION

Although it is not possible to definitively assign bacterial species using 16S rRNA marker gene data, we have demonstrated that it is possible to rule out species assignments for all but a few species. Our software automates this process and computes a rule-out probability for compatible species. Based on the evaluation of full genome vs 16S gene similarity, we showed that our probability model is accurate for most bacterial species (with the notable exception of *Prevotella copri*). Our software is consistent with the existing method implemented in DADA2 but produces a greater number of consistent and high-quality annotations. Unassigner works better for host-associated sites where there is a greater representation of type strains in databases compared to environmental samples. Unassigner performs well across three widely used regions of the 16S genes, though we note that a considerable number of species are indistinguishable in the V4 region, relative to other variable regions in the gene.

Our approach treats taxonomic assignment at the species level as a qualitatively different problem from assignment at higher taxonomic ranks. At the rank of genus and above, it is appropriate to ask whether a query sequence is well-placed within a pre-existing group of reference sequences, as most taxonomic assignment methods do. However, at the rank of species, the type strain plays a more central role in determining membership in the taxon, as evidenced by the practice of microbiologists working to establish new bacterial species in the laboratory. Even though using 16S rRNA gene sequences of the type strains is not guaranteed to reflect similarities of the whole genome, simply adopting taxonomic assignment methods developed for the genus or family level is unlikely to yield good results for bacterial species.

The method presented here addresses a critical perceived weakness of 16S rRNA marker gene sequencing, namely, that it cannot be used to achieve species-level resolution. By providing accurate rule-out probabilities for named bacterial species, we hope to place the taxonomic assignments from 16S marker gene sequencing on more even footing with those obtained in shotgun metagenomic studies. This would provide an advantage in studies where it is not cost efficient to do shotgun metagenomic sequencing or when the samples contain a lot of host reads where 16S rRNA gene sequencing would be more beneficial. Once a researcher identifies the 16S rRNA sequence of an organism of interest, the Unassigner software could provide more specific information on the identity, which allows for more targeted downstream hypothesis testing. Moreover, data about bacterial phenotypes, such as aerotolerance and metabolic products, are commonly cataloged at the species level. A quantitative assessment of sequence compatibility with named bacterial species may facilitate connecting sequence data to phenotypes.

Although our software provides a considerable improvement to existing methods, our work raises additional questions that might be addressed in future publications. First, we could extend the method to allow integration of un-named or unofficial bacterial species. Segmented filamentous bacteria would be a prime candidate for inclusion. Second, our approach could be extended further to help users manage species groups that are essentially indistinguishable by 16S sequence alone and to make connections between results for different 16S gene regions. Finally, we could extend the method to other amplicon targets, such as the internal transcribed spacer, which is often used for marker gene sequencing of fungi.

## MATERIALS AND METHODS

### Reference data set

The Unassigner software automatically downloads the Living Tree Project (LTP) database for use as the reference data set (5, 6). We used the August 2023 version of the LTP database for results presented here.

### Comparison of 16S rRNA gene percent identity to average nucleotide identity

We downloaded high-quality genomes from the type strains of all available bacterial and archaeal species from NCBI RefSeq database. We used the Stackebrandtcurve software, created for this study, to compare 16S rRNA gene similarity to genome-wide ANI for genomes deposited in NCBI RefSeq. We used the software to collect all genomes with 16S gene identity of 90% or greater (the default value for—min_pctid). The software computed ANI values for a maximum of 200 genomes at each unique value of percent identity (the default value is 100 genomes).

### Methods comparison using published microbiome data sets

Paired-end 16S rRNA marker gene sequence data from three previously published human-associated microbiome studies (12–14) were downloaded from NCBI Sequence Read Archive (SRA) using BioProject accessions PRJNA629755, PRJNA473785, and PRJNA518155. Three single-end 16S rRNA marker gene sequence data sets from the Earth Microbiome Project representing soil (16), marine (17), and freshwater (15) biomes were downloaded from the NCBI SRA database using BioProject accession PRJEB42019. The sequence data were processed using QIIME2 version 2023.2.0 (22). After demultiplexing, reads were trimmed to 240 bp and clustered to form ASVs according to DADA2’s denoising function (10). Single-end sequence data were trimmed to 150 bp and processed similarly.

To evaluate the Unassigner software, we calculated rule-out probabilities for each ASV using the default settings (hard-threshold algorithm, constant-mismatch-rate algorithm), as described in Supplemental Methods. An ASV was considered to be compatible with a species if the rule-out probability was less than 0.5. If multiple species were identified as compatible, the species with the lowest rule-out probability was selected for analysis.

To evaluate species-level assignment with DADA2, we used the assignSpecies() function in the DADA2 R package version 1.26. The RDP classifier training set version 18 (23) was used as the reference database. Only unambiguous matches were used.

For each ASV, the assignments from both Unassigner and DADA2 were compared. The discrepancy in naming conventions between RDP and LTP was mitigated by updating the assigned species names to the preferred names in the NCBI Taxonomy database using the Taxonomy name/id Status Report Page online tool (24). They were categorized into “both agree” if the species level assignment was the same using both software, “conflicting species labels” if both software assigned a species but they were not the same, “Unassigner only” and “DADA2 only” if only one software could identify a species when the other one failed to find an appropriate match. From each study, 10 species that account for the greatest number of reads in the study were selected for display in the main figures.

### Comparison of 16S gene regions

The V1-V3, V1-V2, and V4 regions of the 16S gene were extracted from type strain sequences in the LTP database with the Trimragged software (Supplemental Methods), which is included with the Unassigner software. To extract the V1-V2 region, we used the F27 (5′-AGAGTTTGATCCTGGCTCAG-3′) and R228 (5′-TGCTGCCTCCCGTAGGAGT-3′) primer sequences (18). To extract the V1-V3 region, we used the F27 (5′-AGAGTTTGATCCTGGCTCAG-3′) and R534 (5′-ATTACCGCGGCTGCTGG-3′) primer sequences (19). To extract the V4 region, we used the F515 (5′- GTGYCAGCMGCCGCGGTAA-3′) and R806 (5′- GGACTACNVGGGTWTCTAAT-3′) primer sequences (20).

The extracted regions as well as the whole-gene sequences were then aligned against the LTP database with vsearch, using the usearch_global option and setting the penalty to zero for terminal gaps. We considered sequences that aligned with 0 or 1 mismatch to be indistinguishable by marker gene sequencing.

## Data Availability

The main software presented here is available at https://github.com/PennChopMicrobiomeProgram/unassigner. The software to compare 16S gene identity to full-genome identity is available at https://github.com/kylebittinger/stackebrandtcurves. Analysis code used for the manuscript is available at https://github.com/ctanes/unassigner_paper_figures. Data used for the analysis are available at https://zenodo.org/doi/10.5281/zenodo.10883554. The QIIME2 snakemake pipeline used to analyze datasetsdata sets is available at https://github.com/PennChopMicrobiomeProgram/16S_QIIME2.

## SUPPLEMENTAL MATERIAL

Data S1 msystems.00515-24-s0001.csv Data to generate Fig. 2A and B.

Supplemental figures msystems.00515-24-s0002.docx Figures S1 to S6.

Supplemental methods msystems.00515-24-s0003.docx Detailed description of how Unassigner and Trimragged softwares work.

ASM does not own the copyrights to Supplemental Material that may be linked to, or accessed through, an article. The authors have granted ASM a non-exclusive, world-wide license to publish the Supplemental Material files. Please contact the corresponding author directly for reuse.
