# Supplementary material for: Unassigning bacterial species for microbiome studies
Source: mSystems. 2024 Jun 24;9(7):e00515-24. doi: 10.1128/msystems.00515-24 (PMC11264914; doi:10.1128/msystems.00515-24)
Supplement: Supplemental figures — Figures S1 to S6. [file msystems.00515-24-s0002.docx]

**Supplementary Figures**

**Supplementary Figure 1:** Association between average nucleotide identity (ANI) and 16S rRNA alignment percent identity for all the species investigated. Orange represents the genomes that are marked as the same species in RefSeq and blue represents genomes that are marked as a different species. The vertical dashed line indicates the conventional average nucleotide identity threshold to determine species membership.

**Supplementary Figure 2:** A) True positive and true negative rate as a function of 16S gene identity. An ANI threshold of 95% is used as the ground truth for identifying two genomes as belonging to the same species. A true positive is defined as the instances where a pair of genomes also has high 16S rRNA gene identity for the specified identity threshold. A true negative is defined as the instances where the ANI was below the 95% threshold as well as the 16S rRNA gene identity for the specified threshold. True positive rate is defined as the number of true positives over the sum of true positives and false negatives. True negative rate is defined as the number of true negatives over the sum of true negatives and false positives. The dashed line represents the traditional 16S rRNA gene identity threshold to assign a read at species level. B) The area under the curve of the ROC curves for species identification for the 30 bacteria and 3 archaea of interest. C) The distribution of area under the curve values for species identification calculated for all the available bacterial and archaeal type strain genomes in NCBI RefSeq database.

**Supplementary Figure 3:** Percentage of genome pairs that are correctly identified by 16S rRNA gene identity, where the correct answer is determined by ANI between the genomes for all the available bacterial and archaeal type strain genomes in NCBI RefSeq database.

**Supplementary Figure 4:** Rule-out probability distributions obtained from the default algorithm. The colors represent the region of the 16s rRNA gene extracted from the Living Tree Project database.


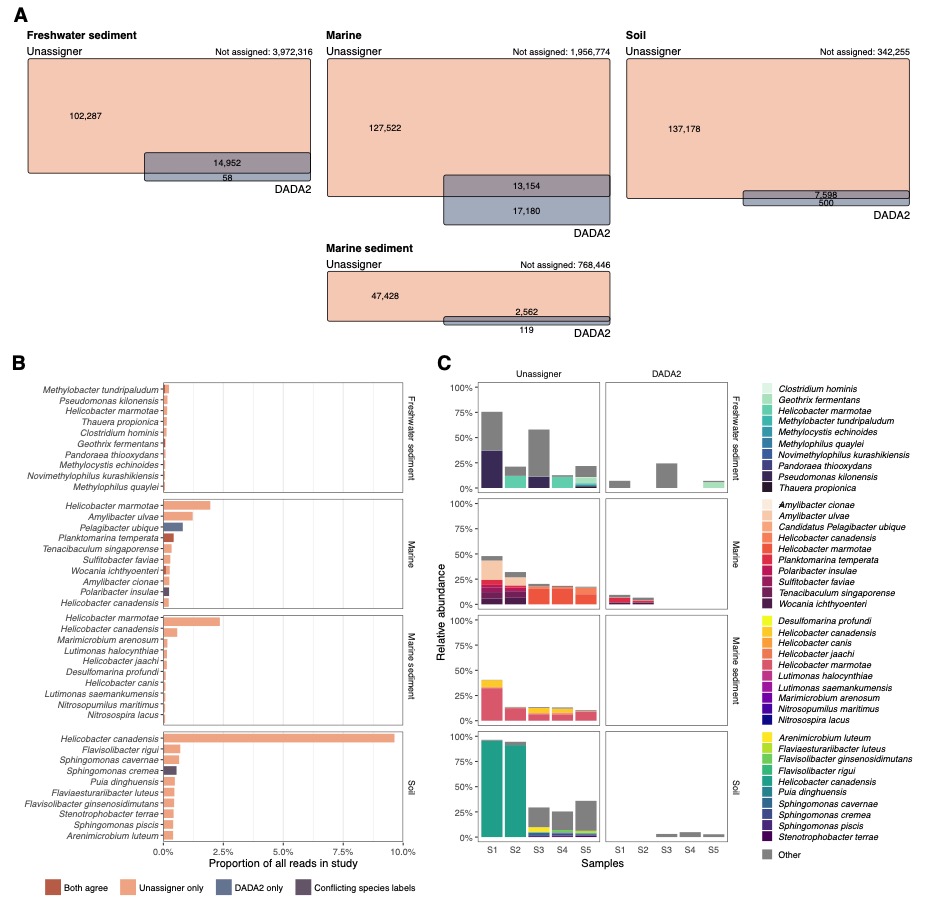


**Supplementary Figure 5:** Comparison to existing methods for environmental samples. Results are shown for three sub-studies from the Earth Microbiome Project for freshwater sediment, marine, marine sediment and soil samples. (A) Venn diagrams of the number of reads assigned to a species with DADA2 or found to be compatible with a species using the unassigner software. The number of reads that could not be attributed to a species by either method is noted above each diagram. (B) Species that account for the greatest number of reads in each study. The colors represent the reads that were annotated exclusively by one method, consistently by both methods and the number that had conflicting assignments between methods. (C) Species composition of select samples. Uncolored regions represent reads without species inference. From each dataset, the five samples with the highest annotation coverage were selected for display.

**
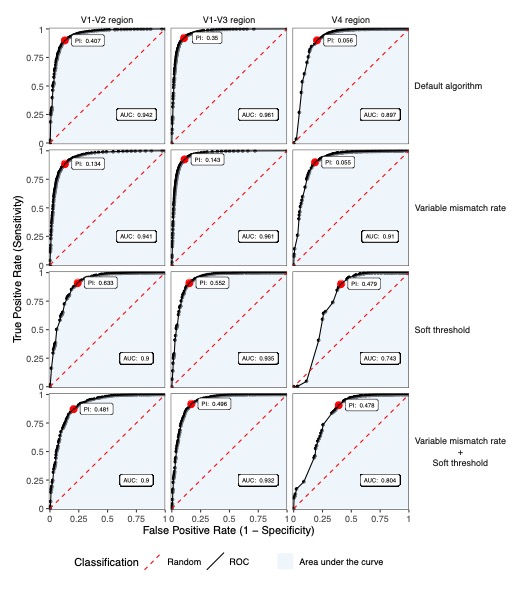
**

**Supplementary Figure 6:** The receiver operating characteristic (ROC) curve showing the sensitivity and specificity of the different algorithms implemented in the Unassigner software. The elbow point is calculated using Youdan’s index as max(sensitivity + specificity -1). The rule-out probability corresponding to the elbow point are marked on the figures. The area under the curve is calculated using the trapezoid method.
