# Supplementary material for: Unassigning bacterial species for microbiome studies
Source: mSystems. 2024 Jun 24;9(7):e00515-24. doi: 10.1128/msystems.00515-24 (PMC11264914; doi:10.1128/msystems.00515-24)
Supplement: Supplemental methods — Detailed description of how Unassigner and Trimragged softwares work. [file msystems.00515-24-s0003.docx]

Supplemental Methods

**Unassigner software**

Here, we describe the operation of the software in detail, and provide a summary of the algorithms that are available in each step. The software operates in three steps: (1) database search, (2) mismatch estimation over the full-length gene, and (3) probability calculation.

**Step 1: database search.** The software requires a reference FASTA-format database of full-length 16S rRNA gene sequences. In the database file, each species should be represented by one full-length gene sequence from the type strain. The FASTA header lines in the database file must contain the species name following the sequence accession, separated by whitespace. If no database file is provided by the user, the software will download and re-format the full length 16S sequences of bacterial type strains from the Living Tree Project.

The user of the software must provide a FASTA-formatted file of query sequences. Typically, the sequences will cover only part of the 16S rRNA gene sequence, but the software will also function accurately with full-length gene sequences.

Database search is carried out by vsearch, which is a dependency of the software. Database search and alignment is carried out using the global alignment algorithm (usearch_global), with the iddef parameter set to 2 to exclude terminal gaps while calculating pairwise identity. The minimum pairwise identity during the database search is set to 90%, which is far below the limit of species-level identity in 16S sequences. The maximum number of results per query sequence is set to 5, meaning that the software considers only the top 5 most similar species that align with more than 90% identity.

**Step 2: mismatch estimation.** For each alignment, the software estimates the total number of mismatches across the full-length 16S gene, generating a probability distribution for each number of mismatches. The number of mismatches in the aligned region, meaning the portion of the global alignment excluding terminal gaps, is determined from the alignment.

The number of mismatches in unaligned regions of the gene, meaning those regions with terminal gaps in the global alignment, must then be estimated. The software uses a beta-binomial model to generate a probability distribution for the number of mismatches, *m*, in the unaligned region: P(*m*) = BetaBin(*m*; α, β). The parameters α and β control the expected rate of mismatches per base pair (μ = α/(α + β)) and the uncertainty in the mismatch rate (the uncertainty decreases as α + β increases).

The software offers two algorithms to determine the parameters α and β: a constant-mismatch-rate algorithm and a variable-mismatch-rate algorithm. For the constant-mismatch-rate algorithm, we assume that the mismatch rate in the unaligned region matches that of the aligned region. Using the Jeffreys prior, α = *m*_aligned_ + 0.5 and β = *ℓ*_aligned_ – *m*_aligned_ + 0.5, where *m*_aligned_ is the number of mismatches in the aligned region and *ℓ*_aligned_ is the length of the aligned region.

It is well-established that the variable regions of the 16S gene vary to different degrees. Thus, we reasoned that it may be possible to improve on the assumption that the mismatch rate in the aligned region matches that in the unaligned region. For example, if the user submitted query sequences that align to a highly variable portion of the gene (such as V1 and V2), we should estimate a lower mismatch rate for the unaligned regions. Conversely, if the user submits query sequences that align to a portion of the gene that is less variable (such as V4), we should estimate a higher mismatch rate in the unaligned regions.

To implement a variable-mismatch-rate algorithm, the software provides an auxiliary program, named count_mismatches. This auxiliary program uses the Green Genes database, a large database of full-length 16S gene sequences to generate training data for adjusting the mismatch rate, and then writes this training data to a file for use with the main unassigner program. The count_mismatches program uses vsearch to perform global alignments between each type strain sequence and other closely related sequences in the large full-length 16S gene database. For each full-length sequence pair, the program records the coordinates of each position in the type strain sequence that does not match to the sequence from the larger reference database. Generation of the training data is a computationally intensive process that takes several hours on current computers as of 2022.

The training data from count-mismatches can then be passed to the main program. To adjust the mismatch rate, the software first determines the coordinates of the full-length type strain sequence where the query sequence was aligned. Thus, the type strain sequence is divided into aligned and unaligned regions based on the alignment with the query sequence. The software then uses the training data to compute α and β parameters in the aligned and unaligned regions of the type strain sequence, based on the other full-length sequences in the training data. For each sequence from the training data, the software then computes the expected mismatch rate (μ = α/(α + β)) for the aligned region and the unaligned region. The mean log ratio of μ_aligned_/μ_unaligned_ from the training data is then used to adjust the expected mismatch rate for the query sequence outside the aligned region. The sum of α and β is not adjusted, to preserve the degree of uncertainty in our estimates based on the length of the query sequence.

**Step 3: Probability calculation.** Having generated a probability model for the number of mismatches between the full-length 16S rRNA gene sequences of the query and the type strain, the software computes an overall rule-out probability for assignment to the species. The overall rule-out probability is computed by summing over the potential outcomes in our probability model for the total number of mismatches. Thus, we require a function to relate the total number of mismatches to a pre-determined rule-out probability. The software offers two algorithms to determine the rule-out probability function: a hard-threshold algorithm and a soft-threshold algorithm.

In the hard-threshold algorithm, we assume that the rule-out probability is 0 for full-length sequences that exceed the threshold, and 1 for sequences that fall below the threshold. The default threshold is 97.5% identity. To compute the overall rule-out probability, we consider all possibilities for the total number of mismatches and simply add the probabilities for scenarios where the identity threshold is exceeded.

In the soft-threshold algorithm, we model the rule-out probability as an exponential function that is 1 when the number of mismatches is 0, and decays as the number of mismatches increases. The function requires one parameter, which sets the level of full-length sequence identity where the rule-out probability decays to half-maximum. From our studies presented in the main manuscript, we determined that an appropriate value for the default parameter is 99.1% identity. Here, the overall rule-out probability is computed by taking the product of the probability distributions for number of mismatches and rule-out probability, then integrating over the range of potential mismatches.

**Trimragged software**

Here we describe the Trimragged software that we used to extract different regions from the full length 16S rRNA gene. The software is included in the unassigner package. The purpose of this auxiliary software is to account for the full length 16S rRNA sequences where only a part of the primer is present in the sequence. This can be due to low quality at the beginning or at the end of a sequence due to limitations of sequencing platforms.

The software operates in three steps: 1) Matching the full length of the primer, 2) Matching the partial primer, 3) Aligning reads to other sequences with a known primer location. The sequence of the primer to search and trim is required for the software. Only one primer is accepted at a time, so the user needs to run the software twice with each primer sequence.

**Step 1:** The software first searches for the full length of the primer sequence. If mismatches are allowed, then the software expands all possibilities of the primer sequence mutations in a list and searches for each. Once a hit is found, the start and end index is stored as a PrimerMatch object.

**Step 2:** If the min_partial argument is greater than 0, the software then searches for partial matches of the primer in the remaining sequences. The software makes a list of all the possibilities of primers, removing nucleotides from the beginning of the sequence till the minimum length specified by min_partial is reached. Then the software searches for each of the possible primer sequences. Once a hit is found, the start and end index is stored as a Primer Match object.

**Step 3:** The last part of the software relies on building a database of the sequences with already identified primer sequences from the previous two steps. Then the rest of the reads are aligned against the database of sequences with known primer locations using vsearch. Once a hit is found, and the positions of the primers are estimated by extending the aligned region.
